# Tissue expression of squamous cellular carcinoma antigen and Ki67 in hepatocellular carcinoma-correlation with prognosis: A historical prospective study

**DOI:** 10.1186/1746-1596-6-121

**Published:** 2011-12-07

**Authors:** Hemda Schmilovitz-Weiss, Ana Tobar, Marisa Halpern, Izhar Levy, Esther Shabtai, Ziv Ben-Ari

**Affiliations:** 1 Gastroenterology Unit, Rabin Medical Center, Hasharon Hospital, Petah Tiqwa and Sackler Faculty of Medicine, Tel Aviv University, Tel Aviv, Israel; 2 Department of Histopathology, Rabin Medical Center, Beilinson Hospital, Petah Tiqwa and Sackler Faculty of Medicine, Tel Aviv University, Tel Aviv; 3 Department of Histopathology, Rabin Medical Center, Hasharon Hospital, Petah Tiqwa and Sackler Faculty of Medicine, Tel Aviv University, Tel Aviv, Israel; 4 Liver Unit, Hadassah-Hebrew University Medical Center, Jerusalem, Israel; 5 Statistical Service, Tel Aviv Sourasky Medical Center, Tel Aviv, Israel; 6 Liver Institute, Rabin Medical Center Beilinson Hospital, Petah Tiqwa and Sackler Faculty of Medicine, Tel Aviv University, Tel Aviv, Israel

**Keywords:** squamous cellular carcinoma antigen, immunostaining, Ki67, hepatocellular carcinoma, TUNEL assay

## Abstract

**Background:**

Squamous cellular carcinoma antigen (SCCA) is overexpressed in hepatocellular carcinoma (HCC) tissue and in sera of HCC patients. Our aim was to assess hepatic SCCA immunostaining in a series of HCCs and to correlate its presence with cell proliferation, apoptosis and clinical outcome.

**Methods:**

Sixty-one HCC patients were included. Liver specimens were obtained either by biopsy (n = 17) or surgically (resection 27, transplantation 17). Immunostaining for AFP, Ki-67, SCCA and TUNEL assay were performed.

**Results:**

SCCA staining was detected in 83.6% of specimens. A statistical significant correlation was found between negative SCCA staining and mortality (p = 0.026) and a higher immunostaining score for Ki67 (p = 0.017). Positive SCCA staining was associated with well and moderate differentiated tumors (p = 0.022). Using multiple logistic regression analysis, Ki67 and TUNEL assay were found to be significant independent predictors of negative SCCA immunostaining. The area under the receiver operator characteristic curve was 0.87. Kaplan-Meier survival analysis revealed a significant difference between the patient group with positive versus negative SCCA immunostaining relating to survival time (p = 0.0106). Cox proportional hazard regression analysis demonstrated that Ki67 immunostaining and liver transplantation or resection were independently associated with mortality.

**Conclusions:**

SCCA is overexpressed in HCC. SCCA status is associated with cell proliferation, apoptosis and survival. SCCA and Ki67 staining can predict survival. Our study results support a potential association of negative SCCA expression with other markers of poor outcome in HCC. More studies are needed to clarify the role of SCCA in HCC and expand the knowledge of the SCCA antigen in HCC patients.

## Background

Hepatocellular carcinoma (HCC) is a major health problem [1,2]. Its incidence is increasing [3] and it has become the leading cause of death amongst cirrhotic patients [4]. The predominant risk factors are chronic hepatitis B and chronic hepatitis C [5]. Llovet et al reported that 80% of HCC cases developed in a cirrhotic liver. Cirrhosis is the strongest predisposing factor [6].

A number of serum markers have been proposed as a method of detecting HCC. However, alpha fetoprotein (AFP), des-c-carboxy-prothrombin (DGCP) and AFP-L3 fraction are not sufficiently accurate to predict its early diagnosis [7].

Dysregulation of the balance between proliferation and cell death represents a pro-tumorigenic principle in human hepatocarcinogenesis. The Ki-67 protein is associated with active cell proliferation and expressed in all phases of the cell cycle, except G0, with the highest expression seen in G2/M.

In a study of patients who had undergone resection for HCC, higher levels of expression of Ki-67 in tumor tissue were found to be associated with a higher tumor grade [8] and early disease recurrence [9]. The balance between death and survival is dysregulated in HCC mainly due to overactivation of anti-apoptotic pathways such as Bcl-XL, Mcl-1, c-IAP1, XIAP or survivin, which are over-expressed in HCC cells [10].

Squamous cellular carcinoma antigen (SCCA) is a member of the high molecular weight family of **ser**ine **p**rotease **in**hibitors (serpins) [11]. High levels have been reported in cancer of the head and neck tissue and other epithelial cancers [12]. It has also been reported to overexpress in HCC tissue and in serum from HCC patients [13]. SCCA has been reported to overexpress in tumoral compared to peritumoral tissue, suggesting a role as a potential marker for histological detection of HCC [14].

The aim of this study was to assess SCCA immunostaining in a series of HCCs of different etiologies and characteristics and correlate its presence with cell proliferation, apoptosis and survival.

## Patients and Methods

### Patients' cohort

This study was simultaneously conducted in two large transplant medical centers in Israel: Beilinson Hospital, Rabin Medical Center, Petah Tiqwa (affiliated with the Sackler School of Medicine, Tel Aviv University, Tel Aviv) and Hadassah-Hebrew University Hospital, Jerusalem. A search was performed in the pathology departments of both hospitals for patients diagnosed with HCC. These patients were followed up at the Liver Institute, Beilinson Hospital or the Liver Unit, Hadassah-Hebrew University Hospital from January 2004 through December 2010, inclusive. Exclusion criteria were either insufficient liver tissue on the biopsy specimen for extra analysis (9) or insufficient clinical data regarding patient outcome (3). These last patients were non-Israeli citizens and either had returned home to their countries after obtaining a diagnosis or had been treated. The survival period was calculated from the point that tissue diagnosis was performed until either the patient's death or termination of follow up (April 2011) (whichever came first). Mortality data were retrieved from the computer system of the Ministry of Interior affairs via the hospital computers.

Serum AFP was measured in all cases. HCC diagnosis was confirmed by the presence of a focal lesion > 2 cm detected by liver ultrasound (US), computed tomography and magnetic resonance imaging, when indicated. Liver specimens, formalin-fixed and paraffin-embedded, used to diagnose HCC were obtained from all patients either by US-guided biopsies or surgically in patients who had undergone resection or transplantation.

### Serum AFP Level

Immunoassay was used to analyze serum sample AFP levels (Roche Diagnostics) using the Modular Analytics E170.

### Histological Evaluation

The SCCA was analyzed by immunohistochemistry in paraffin sections. A score was obtained by assessing entire sections in each case and each nodule in cases of multiple malignant nodules. The highest score was used. For SCCA detection, a novel polyclonal rabbit antibody (Hepa-Ab, Xeptagen, Italy), raised against recombinant SCCA1 and affinity purified on a Sepharose-SCCA1 column, was used.

Epitope mapping studies with SCCA1 fragments obtained by SCCA1 enzymatic digestion or chemical synthesis indicated that the affinity-purified polyclonal antibody recognized several epitopes located in the N-terminal, C-terminal and the central portion of SCCA1 (determined by an enzyme-linked immunosorbent assay (ELISA) and Western blot). The concentration level of Hepa-Ab used was 4 mg/mL. Sections were incubated with primary antibodies for 30 minutes after blocking endogenous peroxidase activity with 3% hydrogen peroxide. The slides were heated in 10 mM sodium citrate in a microwave oven, blocking nonspecific protein binding in normal goat serum. Biotinylated goat anti-rabbit (Dako, Copenhagen, Denmark) was then added for 30 minutes.

Samples were incubated with avidin-biotin-peroxidase and stained with a mixture of 3,30- diamino-benzidine tetrahydrochloride (Dako) and hydrogen peroxide. As a negative control, sections were incubated, omitting a primary antibody. In each case, a diluent or appropriate nonimmune IgG was substituted. Antibody specificity was confirmed using human skin specimens for SCCA as positive controls.

The percentage of stained cells in each specimen was scored on a scale of 0-3: 0 denoted negative staining; 1 - positivity in 1-30% of hepatocytes; 2- positivity in 31-50%; and 3- in > 50%. Distribution of immunoreactivity was noted and classified as diffuse patterns, clustered or scattered cells.

In all cases, SCCA semiquantitative immunoreactivity was independently evaluated by two pathologist experts in the field. Intra and interobserver differences were < 5%. Discordant cases were simultaneously re-evaluated by the two.

In all immunohistochemical analyses, necrotic areas and edges of tissue sections were not included in the counting as to avoid possible false positivity.

### AFP Immunohistochemistry Staining

Immunohistochemistry on paraffin embedded sections with alpha feto protein antibody was performed using a fully automated system (Ventana Benchmark, United States). Sections were pretreated with protease enzymes and incubated with a Rabbit Anti-Human Alpha-1- Fetoprotein antibody (A008 DAKO DK-2600 Glostrup Denmark) in a concentration of 1:300 for 40 minutes.

### Ki-67 Immunohistochemistry Staining

The primary antibody was a mouse monoclonal anti-human Ki-67 antigen (a mindbomb homolog 1 clone). Immunohistochemistry on paraffin embedded sections with Ki-67 was performed using a fully automated system (Ventana Benchmark, United States). Briefly, heat retrieval was standard for 60 minutes. Sections were incubated with Ki-67 (M7240, Dako) and diluted 1:100 in an antibody diluent (00-3118, Zymed, San Francisco, CA).

Only distinct nuclear staining of carcinoma cells was used for scoring via the light microscope, determined semiquantitatively as nil (no immunostaining), low (10% or less immunopositivity) or high (> 10% immunoreactive cells), respectively. Assessment was carried out on the entire tumor represented in the section.

### Terminal Deoxynucleotidyl Transferase dUTP Nick End Labeling (TUNEL) Assay

Apoptotic cell nuclei were identified by applying an ApoTag in situ apoptosis peroxidase detection kit using the TUNEL assay (Intergen Co., New York, NY). This assay is designed to specifically detect the fragmented DNA of apoptotic cells by catalytically incorporating fluorescein-12-2'-deoxyuridine 5'-triphosphate at the 3'-OH DNA ends using the terminal deoxynucleotidyl transferase enzyme, thus forming a polymeric tail. The fluorescein-12-2'-deoxyuridine 5'-triphosphate-labeled DNA can subsequently be directly visualized by fluorescence microscopy. Hepatocytes with nuclear positivity for DNA fragmentation were counted in 50 ± 5 fields (original magnification ×10).

All practices conformed to institutional guidelines for the protection of human subjects.

### Statistical Analysis

Descriptive statistics are given as median, mean and standard deviation (SD) for continuous variables and frequency distribution for categorical variables. Logarithmic transformation was applied to the serum AFP variable due to skewed distribution of the data. Chi-square or Fisher's exact test were used for categorical variables; the two-sample t-test for continuous variables. A multiple logistic regression model was applied to assess parameters independently associated with SCCA. A receiver operator characteristic (ROC) curve was drawn and the area under the curve (C parameter) was used to quantify the predictive ability of the final regression model. The Kaplan-Meier method was used to evaluate survival rate. Differences in survival between groups were analyzed by the Log-rank test. The Cox proportional hazards regression model was also applied. Results are presented as hazard ratios (HR), together with their 95% confidence intervals. Statistical analyses were performed using SAS for Windows 9.2.

## Results

Mean age of the 61 patients included in the study was 63.18 ± 10.60 years; 70.49% were male. Thirty (49.18%) patients were anti-HCV positive, 17 (27.87%) HBsAg positive, 4 (6.55%) admitted alcohol abuse, and the remaining 9 (16.4%) had no identified risk factors. One tumor lesion was found in 55.9% of the patients and > 1 in 44.1%. Mean tumor size was 5.25 ± 3.93 cm. Tumor differentiation was well in 36.0% of patients, moderate in 47.5% and poor in 8.2%. Treatment options included resection (n = 27), liver transplantation (n = 17) and chemoembolisation (n = 37).

### SCCA Immunostaining

**Figure 1:**
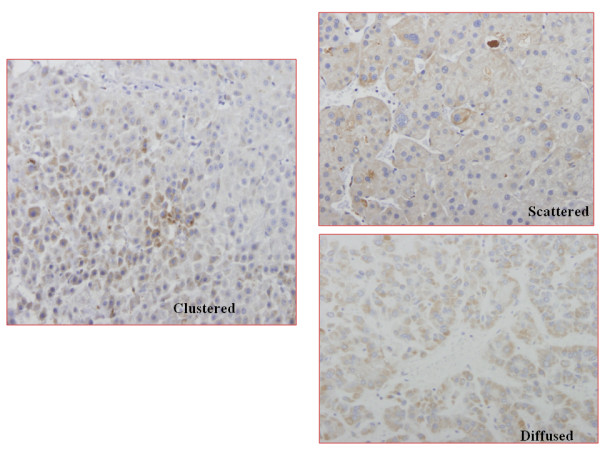
**Pattern of hepatic SCCA immunostaining in HCC**.

SCCA expression in HCC is shown in Figure 1. SCCA was detected in 51 (83.6%) specimens; 17 (27.87%) from transplant specimens; 27 (44.26%) from resection and 17 (27.87%) from fine-needle ultrasound guided biopsies; undetected in 6 surgically obtained normal human livers. A positive signal was clearly detectable in 50% of the cases, with a scored of 1, 19.7% with a score of 2 and 20.3% with a score of 3, Table 1.

**Table 1:** Patients' baseline characteristics (n = 61)

Variable	
Mean age (years)	63.18 ± 10.60
Sex (M/F) (%)	70.5/29.5
Chronic HBV infection (%)	27.87
Chronic HCV infection (%)	49.18
Alcohol (%)	6.60
Other (%)	16.35
One lesion/multiple lesions (%)	55.93/44.07
Mean tumor size (cm)	5.25 ± 3.93
Mean serum AFP level (ng/mL)	4265.24 ± 10672.40
Tumor differentiation:	
Well/moderate/poorly (%)	36/47.5/8.2
AFP immunostaining (positive/negative) (%)	29.27/70.73
Mean Ki67 score	14.42 ± 15.74
Mean TUNEL score	27.67 ± 26.99
SCCA immunostaining (positive/negative) (%)	83.6/16.4
SCCA scoring (%)	
Score 1	50
Score 2	19.7
Score 3	20.3
Liver transplantation (%)	27.87
Hepatic resection (%)	44.26
Chemoembolization (%)	36.39 ± 33.66
Mean survival (months)	36.39 ± 33.66

Abbreviations: M/F = male/female; HBV = hepatitis B virus; HCV = hepatitis C virus; AFP = alpha feto protein; TUNEL = Terminal Deoxynucleotidyl Transferase dUTP Nick End Labeling; SCCA = squamous cellular carcinoma antigen

SCCA was detected in the cell cytoplasm with a prevalent diffuse pattern. In poorly differentiated tumors, a typical clustered coarse pattern was observed (Figure 1). A statistically significant correlation was found between SCCA staining and: 1) mortality: negative SCCA staining was associated with 100% mortality vs a 64.71% in positive SCCA staining (p = 0.026); 2) tumor differentiation: positive SCCA staining was associated with well and moderate differentiated tumors in 95.85% of specimens (p = 0.022); 3) Ki67 immunostaining: negative SCCA staining was associated with a higher Ki67 score (p = 0.017). However, no such correlation was detected in age, sex, etiology of chronic liver disease, tumor diameter and number, serum AFP, AFP and TUNEL staining (borderline), type or level of SCCA staining (Table 2).

**Table 2:** Comparison between 2 groups of HCC patients: positive and negative SCCA immunostaining

Variable	Positive SCAA	Negative SCCA	p value
Age (years)	63.3 ± 10.8	62.4 ± 9.84	NS
HBV infection (positive, %)	27.45	30.00	NS
HCV infection (positive, %)	50.98	40.00	NS
Alcohol (positive, %)	5.88	10.00	NS
Other CLD (positive, %)	74.51	80.00	NS
Serum AFP level (ng/mL)	4853.41 ± 11432.3	736.24 ± 1072.51	0.027
Mean tumor size (cm)	5.13 ± 4.09	6.12 ± 2.71	NS
Tumor number (> 1, %)	84.62	87.88	NS
Tumor differentiation (%)			0.022
Well	37.25	30	
Moderate	52.94	20	
Poor	3.92	30	
TUNEL score	0.24 ± 0.25	0.41 ± 0.30	0.08
Ki67 score	0.10 ± 0.11	0.319 ± 0.22	> 0.0001
AFP immunostaining (negative, %)	60.0	70.7	NS
Liver transplantation (%)	31.37	10.0	NS
Hepatic resection (%)	60.0	54.9	NS
Chemoembolization (%)	60.0	52.94	NS
Survival (months)	39.78 ± 34.78	19.13 ± 20.88	0.026

Abbreviations: HBV = hepatitis B virus; HCV = hepatitis C virus; CLD = chronic liver disease; AFP = alpha feto protein; TUNEL = Terminal Deoxynucleotidyl Transferase dUTP Nick End Labeling

### AFP Immunostaining

AFP staining was negative in 70.7% of the specimens. No correlation was detected between AFP staining and SCCA staining or any clinical parameter.

### Ki67 Immunostaining

Mean Ki67 score was 0.3190 for negative SCCA staining vs. 0.1086 for positive SCCA staining (p = 0.017).

### TUNEL Assay

Mean TUNEL score was 0.411 ± 0.30 for negative SCCA staining vs. 0.249 ± 0.25 for positive SCCA staining (p = 0.08).

### Independent Predictors of Negative SCCA Immunostaining

Ki67 and the TUNEL staining were significant independent predictors of negative SCCA staining. Table 3 represents the multiple logistic regression analysis for these variables.

**Table 3:** Independent predictors of negative SCCA staining

Effect	Point Estimate	95% Wald		p value
		Confidence limits		
Ki67	0.888	0.823	0.958	0.0022
TUNEL	0.957	0.921	0.995	0.0251
HBV	7.172	0.805	63.863	0.0774

Abbreviations: SCCA = squamous cellular carcinoma antigen; TUNEL = Terminal Deoxynucleotidyl Transferase dUTP Nick End Labeling; HBV = hepatitis B virus

**Figure 2:**
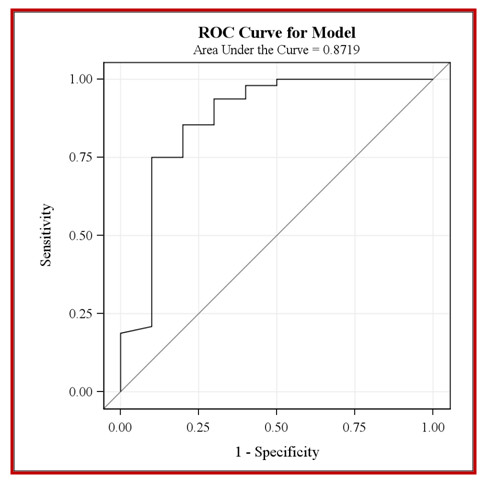
**ROC curve analysis for Ki67 and TUNEL immunostaining in predicting positive or negative SCCA immunostaining**.

ROC curve analysis (Figure 2) revealed that Ki67 and TUNEL staining were excellent predictors for SCCA staining. The C parameter was 0.87.

### Kaplan-Meier Survival Analysis

**Figure 3:**
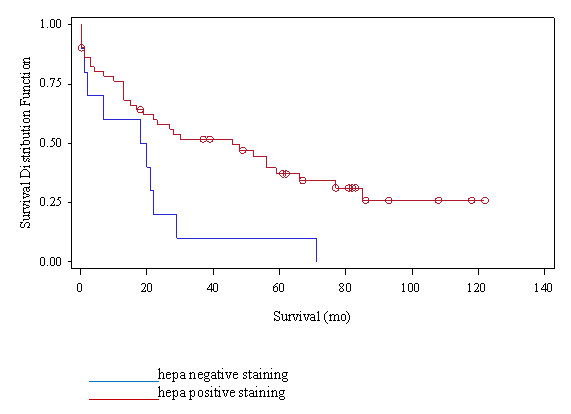
**Kaplan-Meier analysis: A significant difference in survival time between positive and negative SCCA immunostaining groups**.

A significant difference was noted between the two patient groups (positive and negative SCCA staining) in survival time (p = 0.0106) (Figure 3). The mean survival time for negative SCCA staining patients was 19.13 months vs 39.78 months for positive staining patients.

### Variables Independently Associated with Mortality

The following variables were found to be independently associated with mortality: Ki67 staining, HBV, liver transplantation and resection. Table 4 presents Cox proportional hazard regression analysis for each parameter.

**Table 4:** Independent variables associated with mortality

Parameter	Hazard ratio	P value	95% Hazard ratio confidence limits	
Ki67	1.031	0.0092	1.008	1.055
HBV	0.388	0.0205	0.174	0.864
OLT	0.311	0.0151	0.121	0.797
Resection	0.346	0.0117	0.152	0.789
SCCA	0.399	0.0140	0.192	0.830

Abbreviations: HBV = hepatitis B virus; OLT = orthotopic liver transplantation; SCCA = squamous cellular carcinoma antigen

## Discussion

Several studies have reported a high SCCA expression in HCC tissues [13,14]. Giannelli et al [13] measured serum SCCA in 120 HCC patients, 90 cirrhotics, and 41 healthy subjects. SCCA levels were significantly elevated in HCC patients compared to cirrhotic or normal subjects. The sensitivity and specificity for SCCA in HCC diagnosis were 84% and 46%, respectively. Guido et al [15] demonstrated aberrant expression of SCCA in HCC and provided evidence that SCCA overexpression is an early event in liver cell carcinomatous transformation was consistently detected in all considered dysplastic nodules.

The high SCCA expression in HCC tissue seems remarkable since the liver does not possess squamous epithelial cells, although hepatocytes share a common embryogenic origin. Furthermore, the role of serpins in neoplastic cells indicates that SCCA expression makes cancer cells resistant to several killing mechanisms by inhibition of apoptosis [16]. The SCCA antigen represents a marker for more advanced organ tumors such as the cervix, lung and oropharynx [17]. A significant association was also noted between SCCA overexpression and histological grade 2/3 HCC, suggesting SCCA as a marker of cancer aggressiveness [15].

However, in our HCC patient cohort, positive SCCA staining was associated with an improved survival rate. We found that negative SCCA staining was associated with a higher Ki67 score. In multiple logistic regression analysis, the Ki67 and the TUNEL scores were significant independent predictors of negative SCCA staining. A previous study established that Ki-67 immunostaining of HCC lesions was associated with higher mitotic activity [18]. D'Errico et al [9] demonstrated that higher levels of Ki-67 expression in HCC tissue were associated with a higher tumor grade. Nakanishi et al [8] noted that higher levels of Ki-67 expression were associated with early disease recurrence.

Insufficient apoptosis has been associated with the development and progression of liver tumors. [10] In HCC, the balance between death and survival is mainly disrupted due to the overactivation of antiapoptotic signals. Therefore, liver cancer cells might express stronger requirements of these intracellular pathways to survive.

Our study results are in accordance with Trerotoli et al [19] who found that stronger staining was associated with a smaller HCC. They speculated that the higher ratio in smaller rather than larger hepatic nodules, suggests that SCCA is produced and released at different times, likely during the earlier events in HCC progression.

There are several limitations to our study. This is a historical study. Due to the retrospective nature of our study, variable treatment modalities of HCC were included. The patients' cohort was composed of only 61 patients. The group size became even smaller when divided by the SCCA positive and negative staining. Nonetheless, the multiple logistic regression model was applied to assess parameters independently associated with SCCA. The ROC curve was drawn and the C parameter used to quantify the predictive ability of the final regression model.

Up to 40% of all HCC's do not produce AFP [5]. In our study, 70.7% of the specimens did not stain for AFP. No significant correlation was detected between the serum level of AFP, AFP staining and SCCA staining.

Our study results support an invasive diagnostic approach for a suspected malignant hepatic lesion since the SCCA expression in the lesion might provide important information regarding patient prognosis. We feel that if only imaging studies are performed in cirrhotic patients with a suspicious hepatic lesion, valuable prognostic information might be missed.

## Conclusions

SCCA is overexpressed in HCC. SCCA expression is associated with tumor differentiation, cell proliferation and apoptosis. Our study results confirm a potential association of negative SCCA expression with other markers of poor outcome in HCC.

More studies are needed to clarify the role of this molecule and to further expand knowledge of the SCCA antigen in HCC patients.

## Competing interests

The authors declare that they have no competing interests.

## Authors' contributions

HS-W made substantial contributions to the conception and design of the study and was involved in drafting of the manuscript. AT: acquisition of data and interpretation of data; gave final approval of the version to be published. MH: acquisition, analysis and interpretation of data. IL: acquisition, analysis and interpretation of data. ES: made substantial contributions to analysis and interpretation of data. ZB-A: involved in drafting the manuscript and critically revising it for important intellectual content.

All authors read and approved the final manuscript.
